# Macroinvertebrate communities in streams with contrasting water sources in the Japanese Alps

**DOI:** 10.1002/ece3.6507

**Published:** 2020-06-28

**Authors:** Alexander M. Milner, Catherine Docherty, Fredric M. Windsor, Koji Tojo

**Affiliations:** ^1^ School of Geography, Earth and Environmental Sciences University of Birmingham Birmingham UK; ^2^ Department of Biology Shinshu University Matsumoto Japan; ^3^ School of Natural and Environmental Sciences Newcastle University Newcastle upon Tyne UK

**Keywords:** alpine streams, climate change, groundwater, invertebrates, Japan

## Abstract

Alpine streams are typically fed from a range of water sources including glacial meltwater, snowmelt, groundwater flow, and surface rainfall runoff. These contributions are projected to shift with climate change, particularly in the Japanese Alps where snow is expected to decrease, but rainfall events increase. The overarching aim of the study was to understand the key variables driving macroinvertebrate community composition in groundwater and snowmelt‐fed streams (*n* = 6) in the Kamikochi region of the northern Japanese Alps (April–December 2017). Macroinvertebrate abundance, species richness, and diversity were not significantly different between the two stream types. Community structure, however, was different between groundwater and snowmelt‐fed streams with macroinvertebrate taxa specialized for the environmental conditions present in each system. Temporal variation in the abundance, species richness, and diversity of macroinvertebrate communities was also significantly different between groundwater and snowmelt streams over the study period, with snowmelt streams exhibiting far higher levels of variation. Two snowmelt streams considered perennial proved to be intermittent with periodic drying of the streambed, but the macroinvertebrates in these systems rebounded rapidly after flows resumed with no reduction in taxonomic diversity. These same streams, nevertheless, showed a major reduction in diversity and abundance following periods of high flow, indicating floods rather than periodic drying was a major driver of community structure. This conclusion was also supported from functional analyses, which showed that the more variable snowmelt streams were characterized by taxa with resistant, rather than resilient, life‐history traits. The findings demonstrate the potential for significant turnover in species composition with changing environmental conditions in Japanese alpine stream systems, with groundwater‐fed streams potentially more resilient to future changes in comparison to snowmelt‐fed streams.

## Graphical abstract

Macroinvertebrate communities in groundwater and snowmelt fed streams were investigated across the northern Japanese Alps. Unique community structures, and trait characteristics, were influenced by the environmental conditions in the two stream types. Macroinvertebrates inhabiting snowmelt fed streams may be at a greater risk from future climatic change in the region.

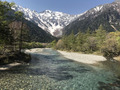

## 1 INTRODUCTION

Alpine streams are fed from a range of hydrological flow paths, including glacial meltwater, snowmelt, groundwater flow, surface runoff, and permafrost melt (Liu, Williams, & Caine, 2004). The variety of water sources generates a mosaic of environmental conditions over a range of spatial and temporal scales (Brown & Hannah, 2008; Fureder, Schutz, Wallinger, & Burger, 2001) thereby allowing for the persistence of an extremely diverse community, which in turn contributes significantly to the regional biodiversity within freshwater ecosystems (McGregor, Petts, Gurnell, & Milner, 1995; Niedrist & Füreder, 2017). Taxonomic heterogeneity across these systems facilitates high diversity in alpine regions, ensuring these regions contribute disproportionately to biodiversity (Hieber, Robinson, Uehlinger, & Ward, 2005; Jacobsen, Schultz, & Encalada, 1997). Climate change and associated hydrological alterations, however, pose a significant threat to alpine streams, many of which are already under pressure from other anthropogenic activities (Brown, Hannah, & Milner, 2007; Finn, Khamis, & Milner, 2013).

Our understanding of alpine stream systems, and those at high latitudes fed by similar water sources and experiencing similar climatic conditions, is relatively well understood across most regions of the world (Europe, North America, Greenland and Svalbard; Blaen, Brown, Hannah, & Milner, 2014; Docherty et al., 2018; Khamis, Brown, Hannah, & Milner, 2016; Windsor, Grocott, & Milner, 2017). However, understanding is more limited in Japan, despite maintaining a unique range of conditions, for example some of the highest levels of snowfall across the world (Ueda, 2014). Furthermore, Japan is a global hotspot for biodiversity, with ~100,000 insect species (Tojo, Sekiné, Suzuki, Saito, & Takenaka, 2017) and high rates of endemism in freshwater fauna (>50%; Balian, Harrison, Butchart, Chambers, & Cordeiro, 2010; Froese & Pauly, 2019; Yoshimura, Omura, Furumai, & Tockner, 2005). As a result, the Japanese archipelago significantly contributes to the biodiversity of aquatic organisms within the east‐Asian subcontinent (Balian et al., 2010), with a large proportion of taxa found in streams across the Japanese Alps (cf. Tojo, Sekiné, Suzuki, et al., 2017). This biodiversity results from streams in this region maintaining a unique range of environmental conditions and thus species assemblages (Balian et al., 2010; Yoshimura et al., 2005).

Climate change predictions for the Japanese Alps include major changes in river discharge due to increased air temperature and rainfall, decreased winter snowfall and spring snowmelt (Sato, Kojiri, Michihiro, Suzuki, & Nakakita, 2013). The potential implications of these changes, however, are poorly understood, although they could lead to more extreme events in alpine environments, for example severe floods and droughts (Ledger & Milner, 2015). And such changes would compound the increasing prevalence of alpine streams running dry at certain times of the year (Beniston, 2012; Chiogna et al., 2016), which is already having significant effects on the structure and function of aquatic macroinvertebrate communities (Heino, Virkkala, & Toivonen, 2009; Piano, Doretto, Falasco, Fenoglio, et al., 2019; Piano, Doretto, Falasco, Gruppuso, et al., 2019; Pinna et al., 2016). Thus, assessing the ecological response of headwater stream systems to future climatic change is urgently required (Bush, Nipperess, Turak, & Hughes, 2012). Further to this, predicting the response of the structure and function of stream biotic communities in Japan to variable environmental conditions is critical to understanding how biodiversity and ecological resilience in this biogeographically unique region of the globe may change into the future.

Here, we aim to understand differences in macroinvertebrate community structure between streams with different water source contributions in order to predict likely effects of climate change into the future. The study was conducted in the Kamikochi region, within the Chūbu‐Sangaku National Park, Nagano Prefecture, Japan. The main river system is the Azusa River into which feed a number of short tributaries with different water sources and, being a National Park, anthropogenic modification is less than other areas of Japan. Samples of the macroinvertebrate communities were collected seasonally at a number of times over a period of one year in six tributary streams dominated principally by groundwater (*n* = 3) or snowmelt (*n* = 3). In addition, associated physiochemical variables were measured to determine their potential significance in driving community composition. Three hypotheses were tested:

1. Macroinvertebrate community structure will be significantly different between groundwater and snowmelt‐fed streams, reflecting differences in environmental conditions.
2. The distribution of macroinvertebrate traits within streams will reflect the variability in environmental conditions within groundwater and snowmelt‐fed streams.
3. Variability in the macroinvertebrate communities of snowmelt streams will be seasonally higher when compared to groundwater streams.

## 2 METHODS

### 2.1 Study sites

The study was completed in the Kamikochi valley within the Chūbu‐Sangaku National Park, Nagano Prefecture, Japan (1,743 km^2^)—a region known as the Hida Mountains or northern Japanese Alps. The National Park is characterized by high mountains with Mt. Tateyama (2,455 m) and Mt. Tsurugidake (2,926 m) at the northern reach and Mt. Norikura (3,026 m) at the southern edge. Kamikōchi valley is approximately 18 km in length, with the average valley floor elevation of 1,500 m. Up to about 12,000 years ago, the River Azusa primarily flowed into the Jinzu‐gawa River system on the Hida‐Takayama southern side of Northern Japan Alps but then, potentially due to a volcanic collapse of Mt. Yake‐dake, the old Azusa River became naturally dammed and started to flow north toward Matsumato (Harayama, 2015). This river catchment, and the geology of the Alps region, supports a biodiversity hotspot at Kamikochi and more generally in the Japanese archipelago (Tojo, Sekiné, Suzuki, et al., 2017; Tojo, Sekiné, Takenaka, et al., 2017).

**FIGURE 1:**
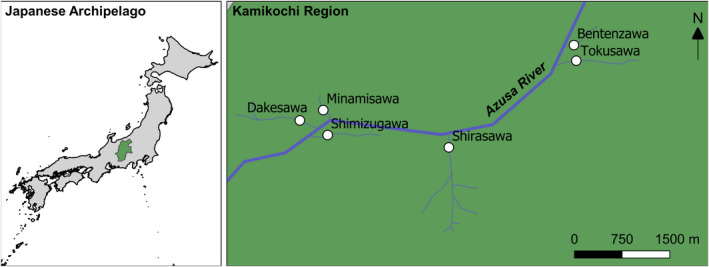
Sample sites at the six streams within Kamikochi

Six streams, initially all considered permanent, were selected in the Kamikochi study area, which were all tributaries of the main Asuza River, three fed by groundwater (Shimizugawa, Minamisawa and Bentenzawa) and three fed predominantly by snowmelt (Dakesawa, Shirasawa and Tokusawa), see Figure 1. Groundwater streams were characterized by stable flow regimes with dense riparian vegetation and negligible variation in water temperature. Streams were typically <5 km in length with mean widths varying from 4 to 10 m and mean depths <300 mm (Table 1). A stream reach of 10 m was designated as the sample site and was typically above any culvert that was used for a road or path crossing. Sites were typically within 200 m of the main Azusa River.

**TABLE 1:** Coordinates and physical characteristics of sample streams

Site	Decimal degrees		Mean width (m)	Mean depth (cm)
	Latitude	Longitude		
Shimizugawa	36.250482	137.639672	9.4	27.0
Minamisawa	36.256573	137.643104	7.5	26.4
Bentenzawa	36.265480	137.690354	4.6	21.8
Tokusawa	36.261673	137.693700	4.2	17.0
Dakesawa	36.253908	137.634887	4.1	14.7
Shirasawa	36.248523	137.670093	3.6	11.2

### 2.2 Physicochemical characteristics

Water temperature was recorded at 30‐min intervals over the sampling period (01/04/2017 to 01/12/2017) using TinyTag dataloggers (TGP‐4017; Gemini Data Loggers Ltd.) at the six study sites. Water level was measured from 03/08/2017 to 01/12/2017 using several types of logger according to availability, including: Levellogger Edge 3001 (Solinst, Georgetown, Canada), dipperLog (Heron Instruments Inc., Dundas, Canada) and Level TROLL 500 (In‐Situ Inc.). Stream discharge (m^3^/s) was estimated at different flows using stage measurements in conjunction with a conservative NaCl tracer dilution method (Tazioli, 2011) and using standard X‐section approaches with a Kenek EU20 (Toyko, Japan) flow‐meter (Herschy, 2009). Water stage was calibrated against each point measurement of discharge to construct a discharge rating curve over the time period. Conductivity was measured at sites when macroinvertebrates were sampled using a hand‐held multi‐parameter meter (YSI 6050000, USA).

Triplicate water samples (125 ml) were collected at the six stream sites during 2017 (Table S1). These samples were frozen at −20°C within 10 hr of collection. Water samples were analyzed for the following ions; Ca, K, Mg, Na, NH_4_, K, Mg, Ca, Si, Cl, NO_2_, NO_3_, SO_4_ using a Hitachi U‐2000 analyser and standard analytical methods for these ions (APHA, 2005).

### 2.3 Chlorophyll *a*

Standardized scrapes of biofilm from three different stones were taken for quantifying chlorophyll *a* at three time periods during 2017. For each replicate, a 5 × 5 cm surface was scraped using a toothbrush and the accumulated slurry washed into a 50 ml vial with stream water. Samples were stored frozen at −20°C. Chlorophyll *a* was measured by extracting in 45 ml of 95% ethanol at 4°C for 24 hr in the dark and reading optical density at 664 nm and 750 nm using a UV‐visible spectrometer (JASCO V‐630, Tokyo, Japan) and then again after adding 0.1 ml of 0.1 N HCL to the cuvette.

### 2.4 Benthic organic matter

Organic matter (in the top 5 cm of the benthos) was collected from Surber samples at each site. After macroinvertebrates were extracted from the samples, organic matter was removed using density separation (Hauer & Lamberti, 2007). Particles were filtered through a 250 µm sieve to collect both fine and coarse particulate organic matter. The extracted organic matter was dried at 60°C for 24 hr to estimate the dry mass of organic matter (OM mg dry weight) for each sample.

### 2.5 Macroinvertebrate communities

Five replicate macroinvertebrate community samples were collected within a 10 m reach at each sample site (*n* = 6) in 2017, from April until October, using a modified Surber sampler (0.093 m^2^, 330 µm mesh). This produced a total of 125 macroinvertebrate samples over all sites and time points (Table S1). Samples were systematically collected from riffle habitats, the dominant habitat type. Macroinvertebrates were preserved in 90% ethanol on site and transported to the laboratory at Shinshu University for sorting and identification. All macroinvertebrates were identified to the lowest practical level (species and genus for most specimens) using available taxonomic keys for Palearctic macroinvertebrates and a Japanese key book (Kawai & Tanida, 2018; Merritt & Cummins, 1984; Wiederholm, 1983). A total of 11,758 macroinvertebrates were identified from 26 families (Table S2).

Trait data for individual macroinvertebrate taxa were collated from trait databases for Palearctic aquatic macroinvertebrate genera (Vieira et al., 2006). Traits of interest were collated from the database, and categorical traits were converted into fuzzy‐coded trait categories based on expert knowledge, with the affinity of traits for individual macroinvertebrate taxa coded following Chevenet, Dolédec, and Chessel (1994). Fuzzy coding was adopted as not all the trait variation for macroinvertebrate taxa can be adequately encompassed by a univariate descriptor. For example, for body form, taxa are unlikely to have characteristics that fall solely within a single category (e.g., cylindrical and flatten) so a multivariate characterization of the trait provides a more suitable method of classifying the affinity of taxa with a range of life‐history strategies. The traits collated for this study were as follows: minimum and maximum body size (mm), body form and structure (cylindrical, flat, case‐bearing, net‐spinning), voltinism (number of generations per year), feeding group (collector, browser, shredder, predator, grazer), aerial dispersal strength (weak, medium, high), attachment mode (e.g., crawler, swimmer), pupation (none, cobbles, mud), flow preference (slow, medium, fast, side‐pools), and habitat affinity (gravel, mud, cobbles, vegetation).

### 2.6 Statistical analyses

Physicochemical (water temperature, dissolved oxygen, total dissolved solids, nitrate and precipitation) and macroinvertebrate (total abundance, species richness, Shannon diversity index) data were investigated using a series of generalized linear models (GLMs; Nelder & Baker, 2006) and generalized linear mixed models (GLMMs; Bolker et al., 2009). Model structure, families, and link functions depended on the variable of interest and are reported in the results. Prior to analyses, data were explored to prevent common statistical problems (Zuur, Leno, & Elphick, 2010). All models were validated following Zuur, Leno, and Smith (2007) and Thomas et al. (2015), by assessing the residual normality using QQ plots, homogeneity of variance determined by plotting the residuals against fitted values and influential observations using Cook's leverage distances.

Temporal variation in physicochemical variables was summarized using coefficients of variation (CVs: *σ*/*µ*) calculated as the mean values for both spot and continuous samples over the sampling period (2017; *n* = 3–5). Differences in the levels of temporal variation in physicochemical conditions between stream types were assessed using GLMs, with the date of collection used as a fixed effect.

Macroinvertebrate community structure and function were investigated through both univariate metrics and multivariate analyses. For GLMMs, community structure was summarized by a range of metrics, including the abundance of macroinvertebrates (*N*), species richness (*R*), and Shannon's diversity index (1/*D*). Site identity was used as the random effect to control for spatial autocorrelation within samples collected from the same sites. Further to the GLMM‐based assessments, multivariate analyses were also completed on the raw community matrix data across sites. To visualize differences in macroinvertebrate community structure, a Non‐metric Multidimensional Scaling (NMDS) was calculated with Bray–Curtis dissimilarity indices and a Wisconsin double standardization to equally weight common and rare taxa (Kenkel & Orloci, 1986). To provide comparable statistical support for observed patterns in the ordination plot, a negative binomial multivariate generalized linear models (M‐GLM), constructed using “mvabund” (Wang, Naumann, Wright, & Warton, 2012), was used to investigate differences in the structure of communities between sites and streams with different intermittency regimes. RLQ and fourth corner analyses (Dray et al., 2014) were also used to understand how the distribution of macroinvertebrate traits varies across sites and flow intermittency regimes within the data.

## 3 RESULTS

### 3.1 Precipitation and hydrology

**FIGURE 2:**
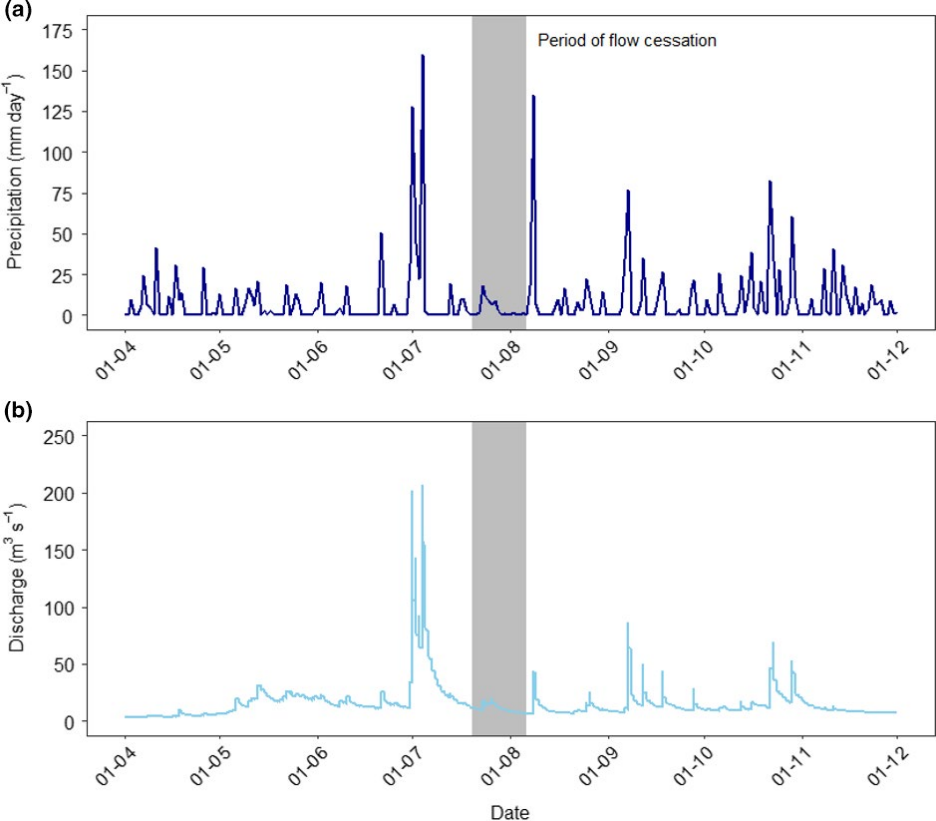
Precipitation and discharge data for Kamikochi Region over the duration of the sampling period. (a) Mean daily precipitation. (b) Mean daily discharge for the main stem of the Azusa River, see Figure 1. The shaded block indicates the period over which flow ceased in both SHIRASAWA and Tokusawa streams

Precipitation was highly variable in 2017 over the sampling period (Figure 2a). For example, there were clear periods of high discharge for the Azusa River corresponding to both snowmelt and intense rainfall (Figure 2b). However, more prolonged periods of low discharge occurred during the summer months (Figure 2b). In particular, the absence of significant precipitation, in conjunction with a limited summer snowpack, during early July to early August 2017 led to the complete cessation of flow in two of the snowmelt‐fed streams, Shirasawa and Tokusawa, previously considered to be permanent. However, peaks in rainfall also occurred during the sampling period, most notably when 355 mm fell on the first 4 days in July (160 mm in 24 hr) and 135 mm in 24 hr on August 8th. There were also significant peaks in early September and later in October (Figure 2a).

### 3.2 Physicochemical characteristics of groundwater and snowmelt streams

Groundwater‐fed streams were characterized by relatively invariant physicochemical conditions, with far lower variation in water temperature, conductivity and discharge (Table 2). Snowmelt‐fed streams, on the other hand, were far more variable in their characteristics and also exhibited far greater levels of seasonal variation in environmental variables. For example, the CVs for stream discharge were particularly different between groundwater and snowmelt streams (Gaussian GLM: *R* ^2^ = .87, *F*_1,4_ = 35.6, *p* = .003), with snowmelt streams exhibiting far higher levels of discharge variation over the sample period (*t*_1,4_ = 5.96, *p* = .004). Water temperature CVs, however, were not significantly different between stream types and there were high levels of variation between different sites with the same source contributions (Gaussian GLM: *R* ^2^ = .11, *F*_1,4_ = 0.6, *p* = .59), and one groundwater‐fed stream, Bentenzawa, had an extremely variable water temperature in comparison to the two other groundwater streams which maintained more stable water temperatures (<1°C variation over a year of measurement). Finally, several of the major ion concentrations exhibited less variation in groundwater streams across the Kamikochi area (Table S3), yet this was not significant.

**TABLE 2:** Environmental variables of streams sampled across the Kamikochi Region (mean plus range. GW = groundwater, SM = predominantly snowmelt)

Site	Flow regime	Mean discharge (m^3^/s)	Mean water temperature (ºC)	Conductivity (µS/cm)	Silica (mg‐SiO_2_/L)	pH	TDS (mg/L)	DO (mg/L)	OM (mg/m^2^)	SSC (mg/L)
Shimizugawa	GW	1.37 (1.03–1.98)	6.5 (6.4–6.8)	103 (37–264)	7.9 (7–8.5)	6.7 (6.3–7.1)	69 (18–205)	11.4 (8.9–14.1)	3.4 (0.3–9.0)	0.005 (0–0.001)
Minamisawa	GW	1.64 (1.35–1.95)	5.6 (5.1–5.9)	118 (9–416)	5.9 (4.8–8)	6.5 (5.5–7.1)	59 (5–208)	11.3 (10.2–12.5)	2.0 (0.8–5.8)	0.0023 (0–0.004)
Bentenzawa	GW	0.47 (0.29–0.66)	6.5 (6.1–7.0)	144 (23–394)	8.1 (7.6–8.6)	7.3 (6.1–9.3)	73 (11–197)	11.9 (11–12.5)	13.4 (2.4–35.5)	0.0022 (0–0.004)
Tokusawa	SM	0.14 (0.1–0.29)	7.7 (5.2–10.4)	128 (36–389)	4.8 (3.3–6.7)	7.4 (6.7–8.3)	70 (18–194)	11.9 (11.2–12.6)	4.1 (0.3–12.2)	0.007 (0–0.021)
Dakesawa	SM	0.7 (0.17–0.61)	8.4 (4.2–13.8)	126 (24–367)	6.2 (5.4–7.7)	6.7 (6.3–7.4)	63 (12–183)	11.8 (10.3–12.8)	6.1 (3.0–9.1)	0.0013 (0.001–0.002)
Shirasawa	SM	0.55 (0.05 – 1.63)	6.8 (4.3–13.7)	104 (29–409)	5.7 (5.2–6.5)	6.7 (6.2–7.5)	63 (14–205)	12 (10.7–12.9)	19.5 (1.7–49.7)	0.001 (0–0.002)

DO, dissolved oxygen; GW, groundwater; OM, organic matter; SM, snowmelt; SSC, suspended sediment concentration; TDS, total dissolved solids.

### 3.3 Concentrations of chlorophyll *a* and benthic organic carbon in groundwater and snowmelt streams

**FIGURE 3:**
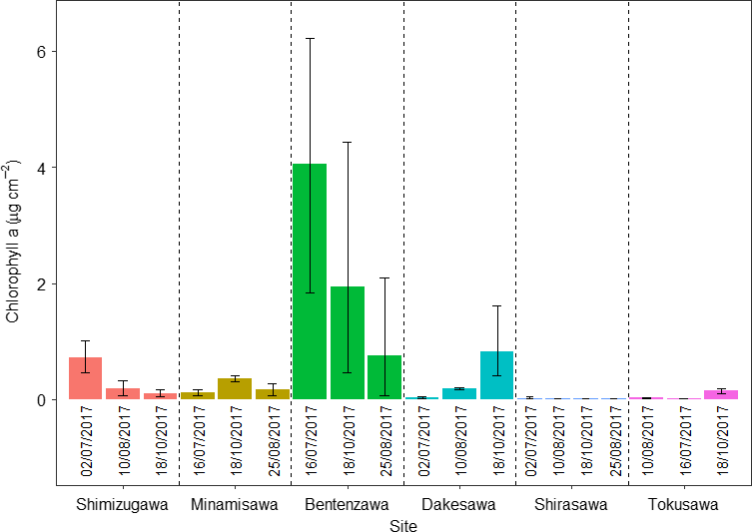
Mean chlorophyll *a* (ug/m^2^) values for the six streams over the study period (July–October 2017)

Chlorophyll *a* concentrations in streams across the Kamikochi Region were highly variable between both streams and sampling periods (Gamma GLM: *R* ^2^ = .43, *F*_5,50_ = 9.24, *p* < .001; Figure 3). Chlorophyll *a* concentrations were significantly lower in snowmelt compared to groundwater streams (*F*_1,54_ = 22.5, *p* < .001), but these concentrations also varied significantly over time (*F*_2,52_ = 6.12, *p* = .004). Chlorophyll *a* particularly showed elevated concentrations in Bentenzawa, compared to the other groundwater streams. Furthermore, stream type and time showed a significant interaction (*F*_2,50_ = 3.73, *p* = .031), with chlorophyll *a* concentration in groundwater streams far less variable over time in comparison to snowmelt streams.

Benthic organic matter was also variable across sites and sampling periods (Gaussian GLM: *R* ^2^ = .37, *F*_9,44_ = 4.42, *p* = .003). Concentrations of benthic organic carbon were not significantly different between groundwater‐ and snowmelt‐fed streams (*F*_1,52_ = 2.53, *p* = .118). Benthic organic carbon concentrations, however, varied significantly over time (*F*_2,50_ = 4.17, *p* = .022) and between individual sites (*F*_4,46_ = 7.02, *p* < .001).

### 3.4 Macroinvertebrate communities in groundwater and snowmelt streams

**FIGURE 4:**
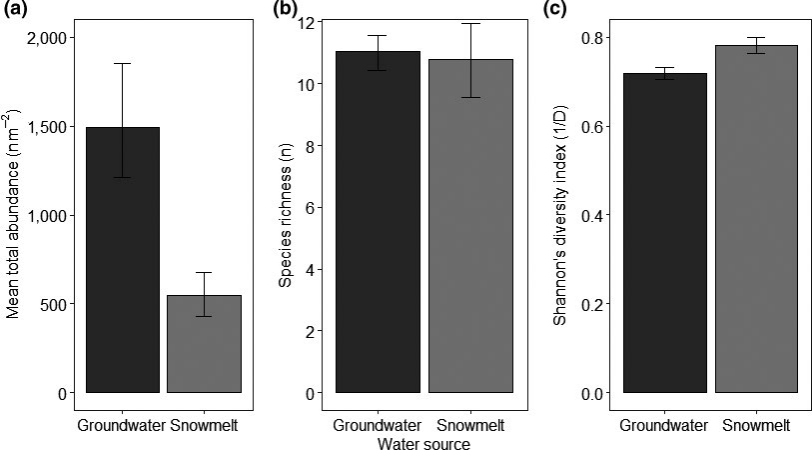
Macroinvertebrate community metrics between the groundwater and snowmelt stream types (± 1*SE*). (a) Mean abundance. (b) Mean species richness. (c) Mean Shannon diversity index

A wide range of macroinvertebrate taxa were observed across groundwater and snowmelt streams (Table S2). Macroinvertebrate community diversity metrics (abundance, species richness, and Shannon's diversity index), however, were not significantly different between the two stream types once models accounted for temporal variation. For example, when stream type, sample period, and their interaction were included in the model for macroinvertebrate abundance (Gaussian GLMM: *R* ^2^ = .57, *F*_5,115_ = 25.08, *p* < .0001; Figure 4), there was no significant difference in total macroinvertebrate abundance between groundwater and snowmelt streams (*t*_1,123_ = −0.31, *p* = .77). For all metrics, potential differences between stream types were masked by high inter‐site variation, which was controlled for using a random effect within GLMMs.

**FIGURE 5:**
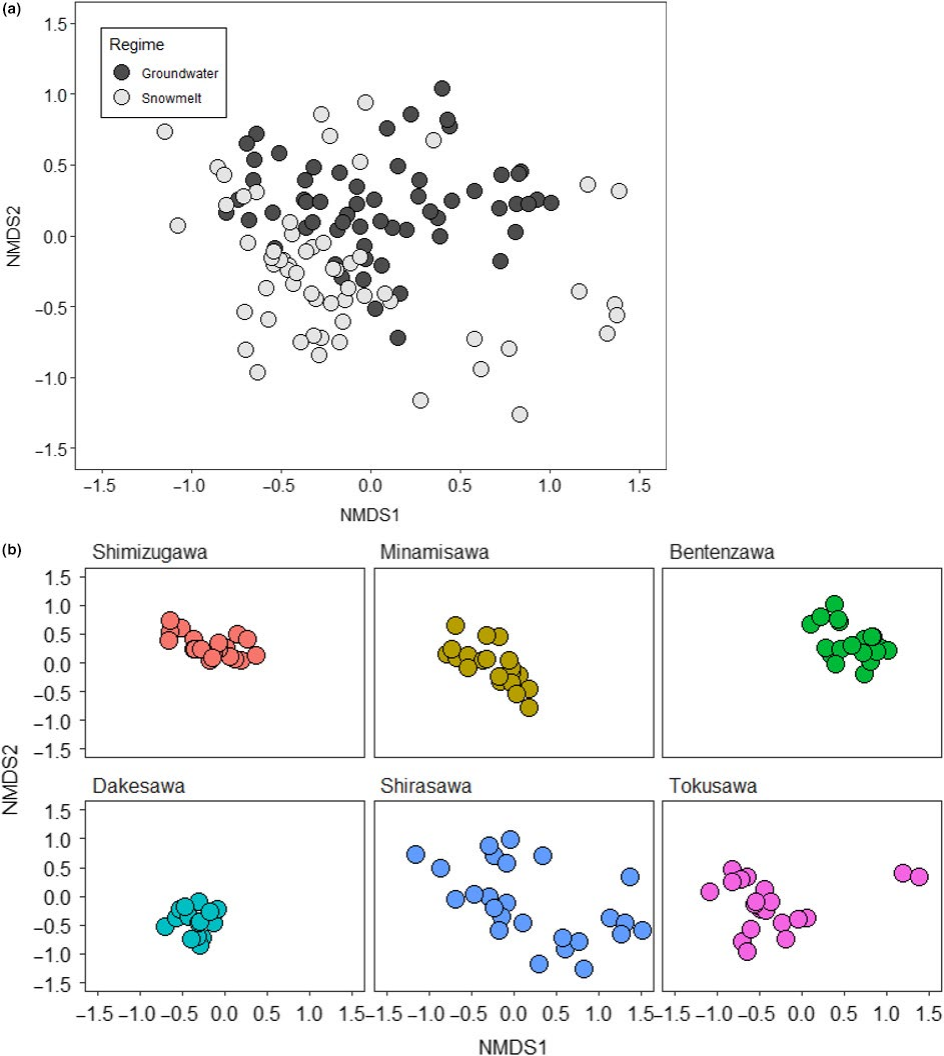
NMDS plot for macroinvertebrate community structure across groundwater and snowmelt streams. (a) Differences between snowmelt and groundwater streams. (b) Differences at the same site over the study period. Groundwater streams are on the top row and snowmelt streams on the bottom row. Individual points represent replicates from each sample unit. Stress = 0.18

Multivariate analyses, however, highlighted significant differences in community structure and species composition between the two stream types (Negative binomial M‐GLM: *R* ^2^ = .11, *W* = 27.38, *p* < .001; Figure 5). Macroinvertebrate taxa in the groundwater streams were a subset of the wider pool of species present across snowmelt streams, with nesting apparent in the NMDS (Figure 5). Several taxa were shown to drive the observed differences between streams. Significantly, there was a higher abundance of several taxa in groundwater streams, including; *Nemoura* sp. (Dev = 24.03, *p* = .004), *Protonemoura* sp. (Dev = 10.95, *p* = .034), *Isoperla* sp. (Dev = 21.87, *p* = .005), *Glossosoma* sp. (Dev = 18.48, *p* = .009), *Megarcys ochtea* (Dev = 13.87, *p* = .034), Chironomidae spp. (Dev = 27.74, *p* < .001), Simuliidae spp. (Dev = 18.42, *p* = .009), and Oligochaetes (Dev = 29.12, *p* < .001). Conversely, *Epeorus* sp. (Dev = 10.14, *p* = .035), *Sweltsa* sp. (Dev = 13.21, *p* = .034), and *Drunella trispina* (Dev = 19.19, *p* = .008) were typically in higher abundance in snowmelt streams.

### 3.5 Macroinvertebrate traits across groundwater and snowmelt streams

**FIGURE 6:**
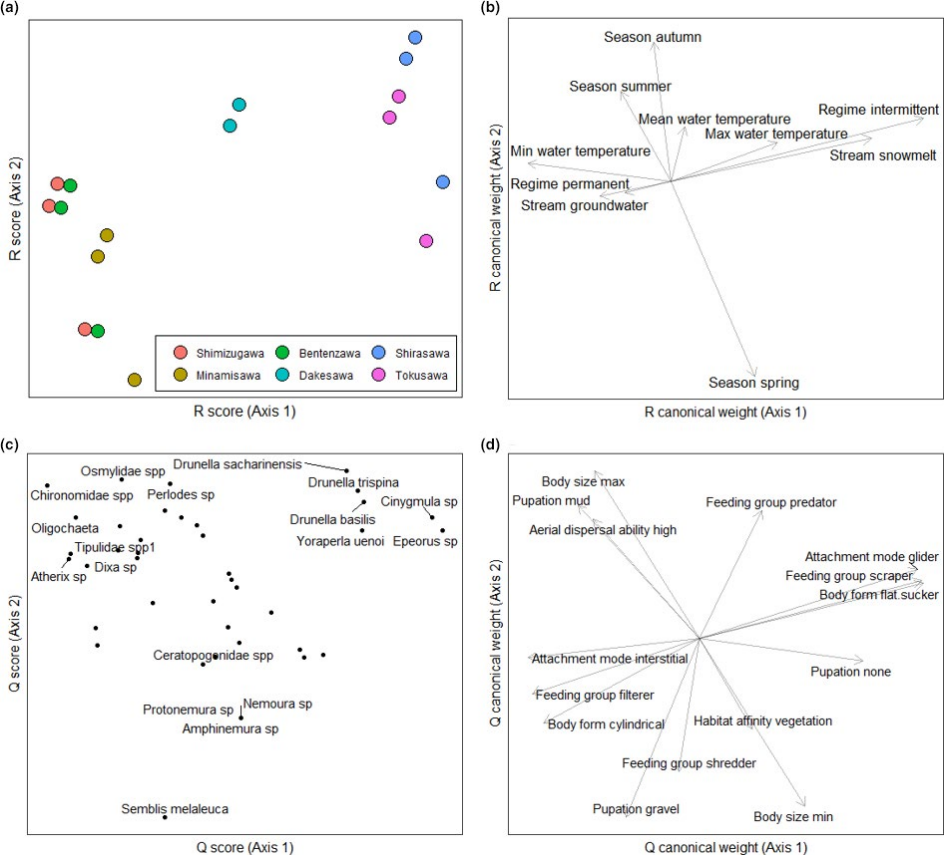
Results from RLQ analyses. (a) *R* scores for samples across the six sample sites. (b) Canonical weights for environmental variables related to variation in the *R* scores for sites. (c) *Q* scores for taxa across sample sites. (d) Canonical weights for macroinvertebrate traits related to variation in the *Q* scores for taxa

The distribution of traits within macroinvertebrate communities varied significantly between sites, with the linkages between the r and q components (through l) appearing significant (Observation = 0.34, *p* = .006; Observation = 0.39, *p* = .004; respectively). A range of taxa and life‐history traits were associated with snowmelt streams across the sample region (Figure 6). Specifically, flat bodied mayflies and flattened stoneflies were found in these systems, which feed predominantly through grazing on epilithic biofilms. Groundwater streams were characterized by taxa with a greater affinity for fine sediments, cylindrical body forms and filter feeding organisms (e.g., Simuliidae) and those making use of silken nets (e.g., *Semblis melaleuca*).

### 3.6 Seasonal variation in macroinvertebrate communities across streams

**FIGURE 7:**
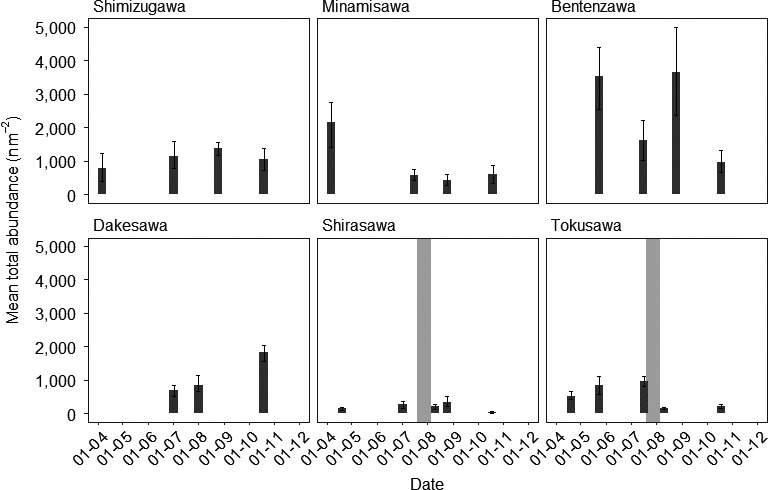
Temporal variation in the mean total abundance per site for macroinvertebrate communities in 2017. Groundwater streams are on the top row and snowmelt streams on the bottom row. Gray shadows indicate periods of no flow in two of the snowmelt streams during the study period

The levels of temporal variation in the abundance, species richness, and diversity were significantly different between groundwater and snowmelt streams (Table 3), with snowmelt streams exhibiting a far higher level of variation (Figure 7). Variation in the community structure, identified using an M‐GLM (Negative binomial M‐GLM: *R* ^2^ = .11, *W* = 27.38, *p* < .001), showed that the season in which samples were collected was more significantly related to changes in community structure than differences between groundwater and snowmelt streams (*W* = 13.04, *p* = .001).

**TABLE 3:** GLM results assessing variation in macroinvertebrate communities

Independent variable	*R* ^2^	Terms	*df*	*t*	*p*
Total abundance (Gaussian)	.27	Flow regime	1, 123	0.55	.48
		Sample period	**2, 121**	**2.89**	**.05**
		Flow regime: Sample period	**2, 119**	**1.95**	**.008**
Species richness (Poisson)	.93	Flow regime	1, 123	0.22	.64
		Sample period	**2, 121**	**7.98**	**<.001**
		Flow regime: Sample period	**2, 119**	**5.42**	**.004**
Shannon's diversity index (Gaussian log)	.58	Flow regime	**1, 123**	**3.99**	**.048**
		Sample period	**2, 121**	**12.83**	**<.001**
		Flow regime: Sample period	**2, 119**	**21.68**	**<.001**

Bold values indicate significant model terms (*p* < .05).

The response of macroinvertebrate communities in two of the snowmelt‐fed streams (Tokusawa and Shirasawa) experiencing unexpected intermittent flow regimes (no flow recorded from July 17th until August 8th in 2017) was inconsistent. Tokusawa also contained no flow on August 25th, and both streams were again dry on December 1st in 2017. Macroinvertebrate abundance in intermittent snowmelt streams significantly varied between sites and over time (Gaussian GLM: *R* ^2^ = .36, *F*_3,46_ = 10.34, *p* < .001). Inter‐site variation in total abundance was significant (*F*_1,48_ = 20.31, *p* < .001) and both sites exhibited large levels of temporal variation (*F*_1,47_ = 6.86, *p* = .012). The patterns of temporal variation, however, were different between the two stream types (*F*_1,46_ = 3.85, *p* = .055), with reductions in total abundance observed in Tokusawa but not Shirasawa (Figure 7). Furthermore, although models for species richness (Poisson GLM: *R* ^2^ = .11, *F*_3,46_ = 3.1, *p* < .001) and Shannon diversity (Gaussian GLM: *R* ^2^ = .16, *F*_3,46_ = 4.11, *p* < .001) including both inter‐site and temporal variation were significant, only Shannon diversity significantly varied over time (*F*_1,47_ = 10.58, *p* = .002) with species richness not significantly variable at either site over the sample period (*F*_1,47_ = 1.43, *p* = .089).

Flows presumably resumed on August 8th, 2017 after 13.5 cm of rain fell in 24 hr following a typhoon (Figure 4). Two days later when macroinvertebrates were sampled on August 10th, species richness was not significantly lower than the July sampling before the intermittency period and even increased in Shirasawa. However, abundance was significantly lower in Tokusawa following the resumption of flow. In October in these two snowmelt streams, Shirasawa and Tokusawa, species richness was reduced to only 2 taxa and no EPT taxa were collected. This reduction followed two major flood events in September 2017. However, Dakesawa, the other snowmelt stream, showed no difference in diversity and actually increased by two taxa than the previous sampling.

## 4 DISCUSSION

Macroinvertebrate communities across streams in the Japanese Alps were diverse and varied in response to a range of environmental characteristics present within the study systems. As one of the few recent studies assessing the structure of benthic macroinvertebrate communities in the streams of the Japanese Alps, data herein provide valuable insights into the potential responses of stream communities to a changing climate. We show that groundwater and snowmelt streams support markedly different macroinvertebrate communities, with high environmental variation in conjunction with flow intermittency in snowmelt‐fed streams, responsible for driving an increased abundance of taxa resistant to high flow variability. In contrast, groundwater streams supported a different suite of organisms more suited to stable flow and higher levels of primary production, as well as organic matter input and retention (filterers and collectors). Differences in the environmental variability and macroinvertebrate community composition, in conjunction with predicted changes in climate and hydrology, mean that groundwater‐ and snowmelt‐fed stream systems may be differentially affected by future climate change.

Environmental conditions in streams across Kamikochi were variable between sites and over time, as is the case for many streams within alpine regions of the globe (Milner, Brittain, Brown, & Hannah, 2010). The climatological conditions in this region, however, are relatively unique with the highest rainfall events in early July and August when other alpine streams across other regions of the globe are relatively low, unless they are fed by glacial meltwater (Brown, Hannah, & Milner, 2003). In the Japanese Alps, the rainy season is typically in July and the typhoon season is between May and October, with the most extreme events in August and September (Yoshimura et al., 2005). These conditions are responsible for generating different hydrological conditions in comparison to other alpine streams in the northern hemisphere. Differences in environmental conditions between snowmelt and groundwater streams, however, followed a highly conserved pattern across many alpine regions—with greater stability of hydrological and physicochemical conditions in groundwater compared to snowmelt‐fed stream systems (Brown et al., 2003; Milner et al., 2010). This stable flow regime is likely responsible for the significantly higher chlorophyll *a* concentrations observed in groundwater‐fed streams. Furthermore, groundwater systems were permanently flowing and did not experience the same extreme events (flooding and flow intermittency) as those present across two of the three snowmelt streams. Extreme flow events, both floods and droughts, can be linked to restricted primary production (as evidenced by the lower levels of chlorophyll *a*), as well as a flushing of benthic organic matter (Boulton & Lake, 1992; Robinson & Uehlinger, 2008).

The first hypothesis H_1_, “Macroinvertebrate community structure will be significantly different between groundwater and snowmelt streams”, was supported. Although abundance, species richness, and taxonomic diversity in groundwater‐fed streams were not significantly different, there were many taxa that were significantly higher between the two stream types. These differences, namely higher abundances of taxa associated with stable conditions in groundwater streams, are likely driven by the aforementioned differences in flow regime and organic matter dynamics, as detritivores are strongly related to organic matter dynamics in streams (Boulton & Lake, 1992). The fact that diversity was not lower in the stable groundwater‐fed streams indicates that competitive exclusion of taxa was not prevalent in these systems. This is likely due to high inter‐patch diversity present within groundwater‐fed streams in Kamikochi. Across the streams, and apparent from the high inter‐replicate variability in Surber samples, the diversity of complex macrophytes, biofilms, bare gravel and cobbles, as well as other habitats, was responsible for generating high macroinvertebrate diversity (at the patch scale) and preventing competitive exclusion through substantial niche segregation (McCreadie & Bedwell, 2013). An alternative explanation may be that the high environmental variability in snowmelt streams reduces the potential biodiversity of these systems, so maintaining a similar macroinvertebrate diversity as groundwater‐fed streams. Certainly, different macroinvertebrate communities were present in groundwater‐ and snowmelt‐fed streams, with snowmelt streams supporting a higher number of unique taxa not found in groundwater‐fed streams, for example, rheophillic mayflies (*Drunella trispina* and *Epeorus* sp.). There was, however, high inter‐site variability within stream types. In particular, Dakesawa on initial selection was considered to be dominated by snowmelt contributions; however, unlike the other two snowmelt‐fed stream systems, Dakesawa was never intermittent and showed markedly less variability in flow and water temperature fluctuation. Therefore, it is likely that the stream received some groundwater inputs during the sample period, and therefore maintained a different set of environmental conditions to those in the other stream systems classified as snowmelt fed within this study. These data further highlight the often‐complex interplay between physical disturbance, site‐specific environmental characteristics and the structure of macroinvertebrate communities in natural systems.

Only specialized macroinvertebrate taxa with a unique subset of life‐history traits were observed in snowmelt streams, likely due to the greater variation in environmental conditions, especially in flow regime. Findings here highlight the unique combination of traits in macroinvertebrate communities occupying snowmelt‐fed streams, in comparison to the broader suite of traits in macroinvertebrate communities across the groundwater streams. This supports hypothesis H_2_ “The distribution of macroinvertebrate traits within streams will reflect the variability in environmental conditions within groundwater and snowmelt‐fed streams.” In this study, it is apparent that taxa inhabiting snowmelt streams are able to persist due to a series of traits enabling individuals to resist disturbances and cope with the unique environmental conditions (e.g., periodic high flows and generally low primary productivity). For example, the greater prevalence of flattened body types and grazing feeding strategies in the snowmelt streams appears to enable macroinvertebrates to persist during seasonal periods of high flows as well as make use of the most abundant resource in these systems, epilithic biofilms.

Although trait analyses indicated that macroinvertebrate taxa appeared to predominantly resist the environmental conditions in snowmelt streams, it is probable that these communities are relatively resilient to both floods and dewatering (e.g., able to recolonize post‐disturbance). The rapid recovery of macroinvertebrate communities after dewatering in the two snowmelt streams (July–August 2017; Figure 7) suggests that taxa with either resistant or resilient strategies allowing for the rapid recolonization of these streams after such extreme events. Two potential explanations appear feasible: (a) that upstream sources of colonizing macroinvertebrates allowed for recolonization of drying stream reaches after rewetting in the case of dewatering, or reduced flows after spates unless the entire stream is affected as likely occurred in October with slow recovery; or (b) that hyporheic refugia is such that either during dewatering or extremely high flows macroinvertebrates are able to avoid adverse conditions through taking refuge in these environments during periods of high disturbance. Few studies have concomitantly compared the resilience and resistance of stream communities to both floods and droughts; thus, it is difficult to draw parallels with other systems. Nevertheless, it is possible that the combination of traits present in the more environmentally variable snowmelt streams enables both resistance and resilience to a range of hydrological disturbances. Certainly, macroinvertebrate communities in upland stream systems with highly variable discharges are not shown to respond to flow variation over intermediate to long timescales (Durance & Ormerod, 2007).

Irrespective of the high inter‐site variation identified between streams across the sample region, the significantly higher temporal variation in the abundance, species richness and diversity of macroinvertebrates in snowmelt streams provides evidence to support H_3_ that “variability in the macroinvertebrate communities of snowmelt streams will be higher seasonally when compared to groundwater streams.” This was particularly marked for streams where flow periodically ceased as a result of low precipitation and snowmelt inputs, which exhibited extreme fluctuations in the abundance, species richness, diversity and structure of macroinvertebrate communities over time. The potential for increasing frequency of such extreme dewatering events into the future therefore poses a significant risk to the biodiversity of these alpine stream systems.

The findings from this study pose a range of interesting questions relating to how streams in the Japanese Alps may respond to future climate change. Due to the hydrological differences between streams in the region, there are a number of likely responses. For snowmelt streams, climate predictions indicate reductions in the levels of snowfall across the Japanese Alps (Sato et al., 2013), which in turn may generate increased incidence of dewatering during the summer months. Furthermore, it is likely that these systems will become more dependent on rainfall, changing both the physicochemistry and hydrology of the systems, and thus altering the composition of the macroinvertebrate communities. As well as changes in snowfall, extreme rainfall events, which also were shown to influence the hydrology of the snowmelt streams, are expected to increase (Ledger & Milner, 2015). For example, over 2019 there were 18 typhoons/extreme tropical storms in Japan which had the potential to generate extreme flood events across the country and these will increase in the future (Normile, 2019). Thus, the changing hydroclimatological conditions predicted for the region present the potential for more frequent dewatering and flood events, placing further pressure on the biological communities in these streams (Tsunematsu, Dairaku, & Hirano, 2013). Groundwater‐fed streams across the sample region are likely to be more resistant to these changes in precipitation, with subsurface flow paths buffering the effects of extreme events and the streams remaining permanent. Groundwater‐fed streams or the upper reaches of stream systems will potentially act as refugia for macroinvertebrate taxa from snowmelt streams during periods of high disturbance. However, it may be the case that the changing environmental conditions across the region result in a loss of several disturbance‐resistant taxa currently inhabiting snowmelt‐fed streams. The full extent of the consequences of these changes for aquatic biodiversity is currently not understood, however, considering current global reductions in freshwater biodiversity such alterations are likely to compound existing pressures placed on these systems.

## CONFLICT OF INTEREST

The authors declare no conflicts of interest.

## AUTHOR CONTRIBUTION

**Alexander M. Milner:** Conceptualization (equal); Investigation (equal); Methodology (equal); Writing‐original draft (equal); Writing‐review & editing (equal). **Catherine Docherty:** Conceptualization (equal); Data curation (equal); Investigation (equal); Methodology (equal); Writing‐review & editing (equal). **Fredric M. Windsor:** Data curation (equal); Formal analysis (lead); Methodology (equal); Software (equal); Visualization (equal); Writing‐original draft (equal); Writing‐review & editing (equal). **Koji Tojo:** Conceptualization (equal); Data curation (equal); Investigation (equal); Methodology (equal); Writing‐review & editing (equal).

## Supporting information

Tables S1‐S3

## Data Availability

Data are stored on Dryad (https://doi.org/10.5061/dryad.wdbrv15kd).
