# Supplementary material for: Macroinvertebrate communities in streams with contrasting water sources in the Japanese Alps
Source: Ecol Evol. 2020 Jun 28;10(14):7812–25. doi: 10.1002/ece3.6507 (PMC7391311; doi:10.1002/ece3.6507)
Supplement: Supplementary file 1 — Tables S1‐S3 [file ECE3-10-7812-s001.docx]

**Table S1. Sample timing and frequency for macroinvertebrates, chlorophyll a and physicochemical spot samples.**

| **Sample type** | **Site** | **Sample dates** | **N** |
| --- | --- | --- | --- |
| Macroinvertebrates | Shimizugawa | 06/04/2017, 02/07/2017,  25/08/2017, 19/10/2017 | 20 |
|  | Minamisawa | 06/04/2017, 16/07/2017  25/08/2017, 19/10/2017 | 20 |
|  | Bentenzawa | 24/05/2017, 16/07/2017, 25/08/2017, 18/10/2017 | 20 |
|  | Tokusawa | 20/04/2017, 24/05/2017, 17/07/2017, 10/08/2017,  18/10/2017 | 25 |
|  | Dakesawa | 02/07/2017, 10/08/2017,  19/10/2017 | 15 |
|  | Shirasawa | 20/04/2017, 02/07/2017, 10/08/2017, 25/08/2017, 18/10/2017 | 25 |
| Chlorophyll a | Shimizugawa | 02/07/2017, 25/08/2017,  19/10/2017 | 9 |
|  | Minamisawa | 16/07/2017, 25/08/2017,  19/10/2017 | 9 |
|  | Bentenzawa | 16/07/2017, 25/08/2017,  18/10/2017 | 9 |
|  | Tokusawa | 16/07/2017, 10/08/2017,  18/10/2017 | 9 |
|  | Dakesawa | 02/07/2017, 10/08/2017,  19/10/2017 | 9 |
|  | Shirasawa | 02/07/2017, 10/08/2017, 25/08/2017, 18/10/2017 |  |
| Physicochemical (spot samples) | Shimizugawa | 06/04/2017, 19/05/2017, 02/07/2017, 25/08/2017, 19/10/2017 | 5 |
|  | Minamisawa | 06/04/2017, 19/05/2017, 16/07/2017, 25/08/2017, 19/10/2017 | 5 |
|  | Bentenzawa | 24/05/2017, 16/07/2017, 25/08/2019, 18/10/2017 | 4 |
|  | Tokusawa | 20/04/2017, 24/05/2017, 10/08/2017, 18/10/2017 | 4 |
|  | Dakesawa | 10/08/2017, 19/10/2017 | 2 |
|  | Shirasawa | 20/04/2017, 19/05/2017, 02/07/2017, 10/08/2017, 25/08/2017, 18/10/2017 | 6 |

**Table S2. Taxa list for macroinvertebrate samples across stream systems.**

| **Taxon** | Shimizugawa | Minamisawa | Bentenzawa | Tokusawa | Dakesawa | Shirasawa |
| --- | --- | --- | --- | --- | --- | --- |
| **Ephemeroptera** |  |  |  |  |  |  |
| Ameletus costalis |  |  |  | * | * | * |
| Baetiella japonica |  | ** | * | * | * | * |
| Baetis bicaudatus |  | * | * | ** | * | * |
| Baetis thermis | * | ** | * | * | ** | * |
| Cinygmula sp. | ** | * |  | ** | * | * |
| Drunella basilis | * | * | * |  | ** |  |
| Drunella sacharinensis | * |  |  |  | * |  |
| Drunella trispina |  | * |  | * | * |  |
| Epeorus sp. |  | * | * | ** | * | * |
| Paraleptophlebia westoni | * |  |  |  |  |  |
| Seratella sp. |  |  |  |  | * |  |
| **Plecoptera** |  |  |  |  |  |  |
| Amphinemura sp. | * | * | * | * | * | * |
| Eucapnopsis sp. |  | * |  |  | * |  |
| Isoperla sp. | * | * | ** |  |  | * |
| Kamimuria sp. |  |  |  | * | * |  |
| Megarcys ochtacea | * | * | ** |  | * | * |
| Nemoura sp. | ** | *** | ** | * | * | * |
| Neoperla sp. |  |  |  | * |  | * |
| Perlinodes sp. |  |  |  |  |  |  |
| Protonemura sp. | ** | * | ** | * |  | * |
| Sweltsa sp. |  | * | * | * | * | * |
| Suwallia sp. | * | * |  | * | * | * |
| Yoraperla uenoi |  | * |  |  |  | * |
| **Trichoptera** |  |  |  |  |  |  |
| Glossoma sp. | ** | * | * | * | ** | * |
| Goerodes sp. |  |  |  |  | * |  |
| Hydrospyche orientalis |  |  |  |  | * |  |
| Lepidostoma sp. | * | * | * | * | * | * |
| Rhyacophilia sp. | * | * | * | * | * | * |
| Semblis melaleuca |  |  | * |  |  |  |
| **Diptera** |  |  |  |  |  |  |
| Antocha sp. |  | * | * |  | ** | * |
| Atherix sp. |  |  |  |  |  | * |
| Blepharacidae spp. |  | * | * | * | * | * |
| Ceratopogonidae spp. |  | * |  | * | * | * |
| Chironomidae spp. | ** | * | *** | ** | * | * |
| Dixa sp. |  |  |  |  |  | * |
| Empididae spp. |  | * |  |  |  |  |
| Osmylidae spp. |  |  | * |  |  | * |
| Simuliidae spp. |  | ** | *** | * | * | * |
| Tipulidae spp. |  | * | * | * | * | * |
| **Coleoptera** |  |  |  |  |  |  |
| Dysticidae spp. | * | * | * | * | * | * |
| **Oligochaeta** | * | * | * | * | * | * |

* = 1-10, ** = 11-49, *** > 50

**Table S3. Major ion concentrations across stream systems. Mean (min–max).**

| **Site** | **Na**  **(mg L^-1^)** | **NH_4_**  **(mg-N L^-1^)** | **K**  **(mg L^-1^)** | **Mg**  **(mg L^-1^)** | **Ca**  **(mg L^-1^)** | **Cl**  **(mg L^-1^)** | **NO_2_**  **(mg-N L^-1^)** | **NO_3_**  **(mg-N L^-1^)** | **SO_4_**  **(mg L^-1^)** |
| --- | --- | --- | --- | --- | --- | --- | --- | --- | --- |
| Shimizugawa | 1.68  (1.12–1.96) | - | 0.3  (0.03–0.5) | 0.49  (0.26–1.07) | 5.56  (3.49–6.73) | 0.34  (0.23–0.5) | 0.001  (0.001–0.002) | 0.16  (0.05–0.19) | 4.08  (2.31–9.35) |
| Minamisawa | 0.97  (0.53–1.43) | - | 0.17  (0.01–0.66) | 0.2  (0.07–1.28) | 1.49  (0.57–4.87) | 0.21  (0.13–0.36) | 0.002  (0–0.002) | 0.19  (0.13–0.32) | 2.03  (1.03–8.57) |
| Bentenzawa | 1.43  (1.21–1.73) | 0.07 | 0.22  (0.05–0.32) | 0.48  (0.45–0.51) | 6.43  (5.69–6.7) | 0.3  (0.25–0.39) | 0.001  (0.001–0.002) | 0.15  (0.13–0.16) | 3.66  (3.47–3.88) |
| Tokusawa | 1.58  (1.28–2.02) | 0.01 | 0.13  (0.02–0.35) | 0.48  (0.38–0.55) | 7.24  (5.56–8.3) | 0.34  (0.24–0.71) | 0.001  (0–0.002) | 0.1  (0.06–0.17) | 3.82  (3.26–4.5) |
| Dakesawa | 1.29  (0.6–1.94) | - | 0.33  (0.17–0.48) | 0.33  (0.15–0.41) | 3.38  (1.61–4.53) | 0.33  (0.19–0.63) | 0.001  (0–0.003) | 0.11  (0.04–0.18) | 5.4  (3.07–7.2) |
| Shirasawa | 1.18  (0.53–1.75) | - | 0.19  (0.05–0.34) | 0.25  (0.1–0.32) | 3.88  (2.02–4.9) | 0.32  (0.18–0.78) | 0.001  (0–0.002) | 0.11  (0.03–0.2) | 3.09  (1.4–3.82) |
